# Thirst

**Published:** 1900-06-02

**Authors:** 


### Thirst.

Recent events in South Africa have drawn attention very strongly to the dangers connected with the drinking of polluted water, while they have also served to show how impossible it is to improvise a safe supply for large bodies of men upon the march. Experience in the same part of the world has also brought forcibly before us a fact which we, living in temperate climates, are apt to forget, namely, that there is no natural impulse so uncontrollable, nothing which dominates men so absolutely, as the craving for water which is produced by a consuming thirst. Preach as we will on sanitary questions, teach our soldiers as carefully as we like that muddy water or water that has been exposed to risk of pollution must at every sacrifice be rigidly avoided, in practice all these maxims are thrown to the winds. In the presence of thirst all men are equal, and we see the educated and the uneducated alike obeying one common and irresistible impulse which drives them to drink the poison which a little later lays them low with typhoid and dysentery. Of all measures for the prevention of disease among troops, by far the most effectual would be the prevention of thirst, if that were possible, and we need not be surprised that, both for this reason and because thirst is one of the most miserable of tortures, inquiries are continually made as to how it can be best prevented, and how best it can be relieved.

In seeking a solution to this important problem the first thing to remember is that thirst is not a mere desire of the lips or a mere craving of the stomach. Thirst is a want arising in every corner of the organism from every tissue being deprived of some of the water which is an essential element in its normal constitution. Putting on one side then such trivial methods as pebble sucking, spice eating, and so on, which act merely by preventing dryness of mouth, real thirst can only be relieved by water, and in whatever form the drink is taken by which it is alleviated it is the water which it contains, and the water alone, that is effectual. Again, the only way to prevent thirst is to avoid undue loss of the watery constituent of the blood. Practically, then, the problem is reduced to this, how best to prevent undue loss of water from the system?

Over the loss of water by the lungs we have no control. Over that by the skin we can exert some influence by care as to clothing and as to drink. The more men drink the more they perspire; and if they obey their natural impulses in this regard, they are sure to lose far more water through the skin than is at all necessary. It is the loss of water by the kidneys, however, that is the most under a man's control, and what we wish especially to insist upon is that alcoholic drinks in consequence of their diuretic action are very ineffectual thirst-quenchers. They relieve for a time, but by their action on the kidneys they remove almost as much fluid as they add, so that the condition of the blood is very rapidly brought back to what it was before, or even made worse than ever. Universal experience enforces the lesson that, however comforting at the time, alcohol produces thirst, especially when much physical exertion is being made. It takes out as much as it puts in, and certainly should not be drunk when economy in fluid is essential. Another and most important cause of wasteful excretion of fluids from the body is impatience of the earlier and bearable degrees of thirst. The human body must part with a certain quantity of water to form the necessary excretions. Nature, however, with her usual liberality has arranged for the watery fluids required for this purpose to be secreted in far greater quantity than is absolutely necessary, and much of this excess of secretion ceases when less fluid is taken into the system. Unfortunately the same water-starvation which diminishes excretion also produces a sense of thirst, and many a man who is impatient of this discomfort and tries to check it by constant sipping might as well empty his water-bottle on to the road. No man can exert himself in a hot country without becoming parched, and this early thirst must be put up with when the water supply is limited. Any attempt to really slake it merely leads to a wasteful passage of fluid through the system.

Then about tobacco. Smoking is probably not in essence such a thirsty proceeding as some people imagine. Habit has much to do with the drinking with which it is often accompanied. Still, tobacco should not be indulged in during the early hours of the march. There can be no doubt that it enables men to bear fatigues and discomforts which without it would be found almost unendurable, and among other things to put up with the miseries of thirst. But the very efficacy of tobacco for this purpose makes it all the more desirable to keep it back until it is really wanted. Practically, however, the two great things which people can do to prevent thirst are not to take alcohol, and to avoid endeavouring by constant sipping to relieve that moderate degree of thirst which of necessity accompanies the natural processes by which excessive and wasteful loss of water from the system is prevented. If a man unscrews his water bottle as soon as he begins to be thirsty he will undoubtedly find it empty long before the great misery comes on. If on the other hand he will put up with the discomfort in its minor stages he may perchance have something left to meet his great need, and be saved from the desperate craving which is produced by real and urgent thirst.

